# HIV Vpr protein upregulates microRNA-122 expression and stimulates hepatitis C virus replication

**DOI:** 10.1099/vir.0.000169

**Published:** 2015-08

**Authors:** Milin Peng, Xinqiang Xiao, Yan He, Yongfang Jiang, Min Zhang, Feng Peng, Yi Tian, Yun Xu, Guozhong Gong

**Affiliations:** Department of Infectious Diseases, Second Xiangya Hospital, Central South University, Changsha, Hunan 410011, PR China

## Abstract

Human immunodeficiency virus (HIV)/hepatitis C virus (HCV) co-infection is characterized by higher serum HCV RNA loads compared with HCV mono-infection. However, the relationship between HIV and HCV replication remains to be clarified. HIV Vpr has been shown to play an essential role in HIV replication. In this study, we aimed to explore the role of Vpr in HCV replication and pathogenesis. We therefore used the genotype 2a full-length HCV strain JFH1 infection system and the genotype 1b full-length HCV replicon OR6 cell line to analyse the effects of Vpr on HCV replication. We found that Vpr promoted HCV 5′ UTR activity, HCV RNA replication and HCV protein expression in two HCV infection cell models. Additionally, lymphocyte-produced Vpr significantly induced HCV 5′ UTR activity and HCV replication in hepatocytes. We also found that Vpr upregulated the expression of miR-122 by stimulating its promoter activity. Furthermore, an miR-122 inhibitor suppressed the Vpr-mediated enhancement of both HCV 5′ UTR activity and HCV replication. In summary, our results revealed that the Vpr-upregulated expression of miR-122 is closely related to the stimulation of HCV 5′ UTR activity and HCV replication by Vpr, providing new evidence for how HIV interacts with HCV during HIV/HCV co-infection.

## Introduction

Human immunodeficiency virus (HIV) accelerates the progression of human hepatitis C virus (HCV)-related liver disease in HIV/HCV co-infected patients (Balagopal *et al.*, 2008; Benhamou *et al.*, 1999; Soto *et al.*, 1997; Thein *et al.*, 2008). In general, the mean time from HCV infection to development of cirrhosis is about 30 years in mono-HCV-infected individuals, but in HIV/HCV co-infected patients the time of onset becomes significantly shorter (Benhamou *et al.*, 1999; Macías *et al.*, 2009; Mohsen *et al.*, 2003; Pineda *et al.*, 2007; Soto *et al.*, 1997) and the occurrence of cirrhosis becomes more frequent (Bonacini *et al.*, 2001; Ioannou *et al.*, 2013; Mohsen *et al.*, 2003; Parodi *et al.*, 2007) than in mono-HCV-infected patients. However, the underlying mechanisms of the accelerated progression to liver fibrosis and cirrhosis due to HIV/HCV co-infection are not fully understood. Previous studies have found that the serum HCV RNA loads are significantly higher in HIV/HCV co-infected patients than in HCV mono-infected patients, strongly suggesting that HIV promotes HCV replication (Eyster *et al.*, 1994; Matthews-Greer *et al.*, 2001). Additionally, some reports have shown that HIV can infect liver cells (Iser et al., 2010a; Steffan *et al.*, 1992). However, no evidence of HIV replication in hepatocytes has been found (Lin *et al.*, 2011; Park *et al.*, 2014), indicating that the acceleration of HCV replication may not involve HIV replication in hepatocytes. Instead, HIV viral proteins may play important roles in promoting HCV replication, as it is believed that Tat (Frankel & Pabo, 1988; Qu *et al.*, 2012) and Nef (Park *et al.*, 2014) could be secreted from the HIV-infected cells and then diffuse into hepatocytes to regulate HCV replication. In addition, it is also known that gp120 can enhance HCV persistence through the upregulation of TGF-β1 expression (Lin *et al.*, 2008).

HIV Vpr is a multifunctional protein with the ability to mediate many processes to activate HIV-1 infection, evade the immune system, and induce viral persistence (Di Marzio *et al.*, 1995; Kogan & Rappaport, 2011). Vpr is a circulating protein that has been found in the serum and spinal fluid of HIV-infected patients (Levy *et al.*, 1994), and can be transduced into a broad array of cells to mediate certain effects of the HIV infection effects on uninfected bystander cells (Sherman *et al.*, 2002). Thus, the questions of whether and how Vpr, as an active protein of HIV, exerts its effect of increasing HCV replication in HIV/HCV co-infection are explored in this study. Recently Deng *et al.* (2014) reported that Vpr increases HCV replication, but the mechanism still needs further investigation.

MicroRNA (miRNA), a small RNA molecule, acts on the post-transcriptional regulation of gene expression and may regulate virus replication. In fact, a close relationship between miRNAs, such as miR-34a, and Tat-induced HIV-1 long terminal repeat (LTR) transactivation has been proved (Zhang *et al.*, 2012). In addition, miR-122 is recognized as an essential mediator of HCV replication via its direct binding to the 5′ untranslated region (5′ UTR) of the viral genome (Jopling *et al.*, 2005; Randall *et al.*, 2007), and a previous study has shown that the expression of miR-122 is significantly upregulated in HIV-infected T-cell lines (Triboulet *et al.*, 2007). However, there is no evidence regarding whether Vpr affects HCV replication by influencing miR-122 expression.

In this study, we sought to explore whether Vpr modulates HCV replication by targeting the HCV 5′ UTR, and then investigate the role of miR-122 involvement in Vpr and HCV replication. Our findings help elucidate one of the mechanisms by which HIV regulates HCV replication and provide new insights into the relationships among HIV Vpr, miR-122, and HCV replication.

## Results

### Vpr activates infectious Japanese fulminant hepatitis 1 (JFH1) replication

**Fig. 1.**
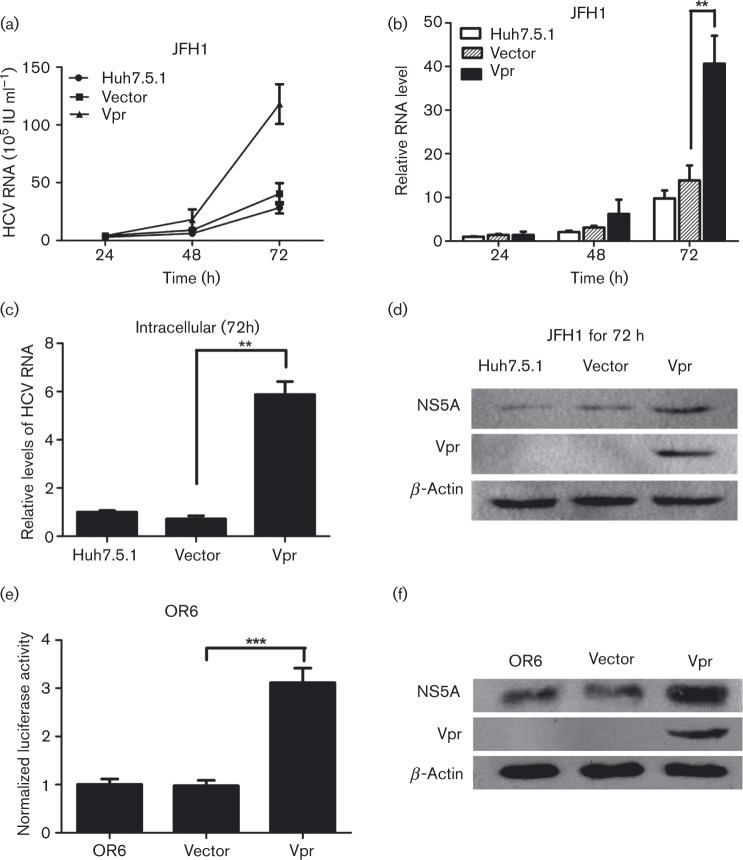
Vpr stimulates HCV replication in JFH1 infection model and OR6 cell lines. (a) Quantitative HCV RNA assay in cell culture supernatants from Huh7.5.1 cells transfected with Vpr and then infected with JFH1 at different times. (b) The relative fold change of HCV RNA levels in cell culture supernatants from Huh7.5.1 cells was measured, with the control non-transfected JFH1-infected Huh7.5.1 cells at 24 h being assigned a value of 1. (c) Quantitative PCR for intracellular HCV RNA levels in the transient Vpr or vector-transfected Huh7.5.1 cells at 72 h post-JFH1 infection. (d) Western blotting confirmed the expression of β-actin, NS5A and Vpr in Huh7.5.1 cell lines infected with JFH1. (e) *Renilla* luciferase assay in OR6 cell lines transfected with Vpr and controls. (f) Western blot analysis in OR6 cell lines confirmed the expression of β-actin, NS5A and Vpr. The data are presented as mean ± sd. **P < 0.01, ***P < 0.001.

To establish the relationship between Vpr protein and HCV replication, we used the JFH1 cell culture model, an established HCV (genotype 2a) infectious cell culture model. Huh7.5.1 cells were first transfected with pcDNA-Vpr or pcDNA3.1, and then infected with JFH1. The cell culture supernatants were collected and the HCV RNA levels were detected at different times. The results revealed, as shown in Fig. 1(a, b), that, in the presence of Vpr, the HCV RNA levels were prominently elevated in a time-dependent manner, reaching a 1.14-, 2.97- and 4.15-fold increase, compared with the levels in the vector-transfected cells (*P* < 0.01), at 24, 48 and 72 h, respectively. We also evaluated the intracellular HCV RNA levels in transiently Vpr-transfected JFH1-infected cells, by measuring total cellular RNA collected at 72 h post-transfection. The results showed that the intracellular HCV RNA levels were significantly increased in the cells transiently transfected with Vpr, reaching a 5.88-fold increase compared with vector-transfected cells (*P* < 0.01), as shown in Fig. 1(c). Consistently, we found that, compared with vector-transfected control cells, Vpr strongly increased the expression of the HCV NS5A protein in JFH1-infected cells (Fig. 1d).

### Vpr increases full-length HCV replicon replication

To investigate the effect of Vpr on HCV replication, we used OR6 cells, which harbour the full-length HCV replicon RNA (HCV genotype 1b) and co-express *Renilla* luciferase as a reporter. The *Renilla* luciferase activity, which reflects the effect of Vpr on HCV replication, was measured. These measurements revealed that Vpr significantly enhanced the relative luciferase activity (3.12- versus 0.97-fold, *P* < 0.001), compared with the vector-transfected cells, as shown in Fig. 1(e). We also assessed HCV NS5A protein expression in OR6 cells and found that, compared with the vector-transfected cells, the HCV NS5A protein expression was significantly increased in the presence of Vpr, as shown in Fig. 1(f). These data indicated that Vpr promoted not only HCV RNA replication but also its protein expression.

### Vpr activates HCV replication in stable Vpr-expressing cells

**Fig. 2.**
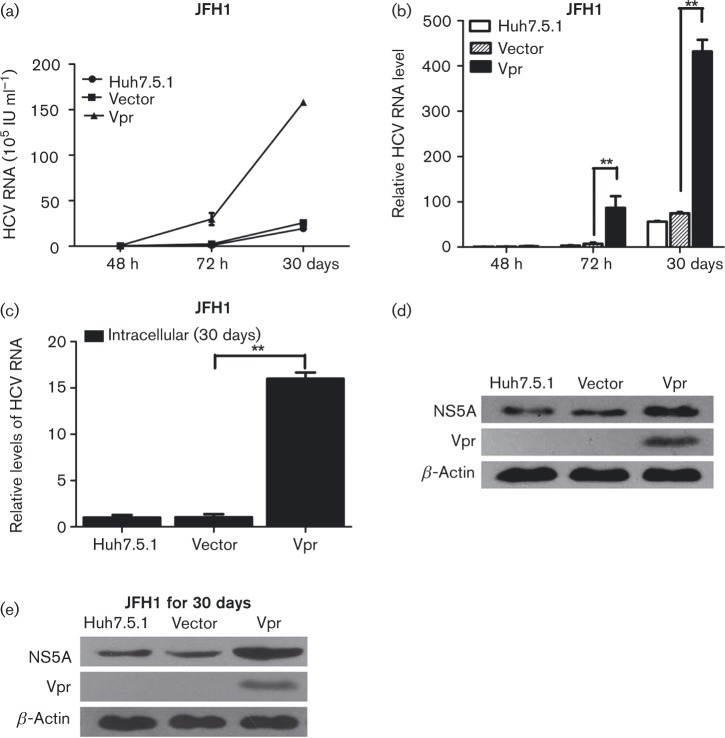
Vpr activates HCV replication in stable Vpr-expressing cells. (a) Quantitative HCV RNA assay in stable vector-Huh7.5.1, Vpr-Huh7.5.1 and Huh7.5.1 cells at 48 h, 72 h and 30 days post-JFH1 infection. (b) Relative fold change of HCV RNA levels. (c) Quantitative PCR for intracellular HCV RNA levels in the stable vector-Huh7.5.1, Vpr-Huh7.5.1 and Huh7.5.1 cells at 30 days post-JFH1 infection. (d, e) Western blotting confirmed the expression of β-actin, NS5A and Vpr in stable Huh7.5.1 cell lines at 72 h (d) and 30 days post-JFH1 infection (e). The data are presented as mean ± sd. **P < 0.01.

We next examined the HCV replication in stably transfected Vpr-Huh7.5.1 or vector-Huh7.5.1 cells infected with JFH1. The Vpr-Huh7.5.1, vector-Huh7.5.1 or Huh7.5.1 cells were seeded into six-well plates and then infected with JFH1. Cell culture supernatants were collected at 48 h, 72 h and 30 days post-JFH1 infection, and the viral RNA was measured at each time point. As shown in Fig. 2(a, b), Vpr markedly enhanced HCV replication in Huh7.5.1 cells stably expressing Vpr, reaching a 1.76-, 12.10- (*P* < 0.01) and 6.15-fold (*P* < 0.01) increase compared with the levels of viral RNA in the vector-transfected Huh7.5.1 control cells, at 48 h, 72 h and 30 days post-infection, respectively. The intracellular HCV RNA level in Vpr-Huh7.5.1 cells at 30 days post-JFH1 infection was significantly enhanced compared with the vector controls (16.00- versus 1.04-fold, *P* < 0.01, Fig. 2c). The expression levels of HCV proteins were also examined in the infected cells at 72 h and 30 days post-JFH1 infection. The expression levels of the HCV NS5A were found to be increased in the Vpr-Huh7.5.1 cells, compared with the vector controls, at 72 h (Fig. 2d) and 30 days (Fig. 2e) post-JFH1 infection. These data strongly demonstrated that Vpr could upregulate HCV RNA replication and HCV protein expression in the early and stable stages of infection of JFH1 cell lines.

### Vpr enhances the HCV 5′ UTR activity

**Fig. 3.**
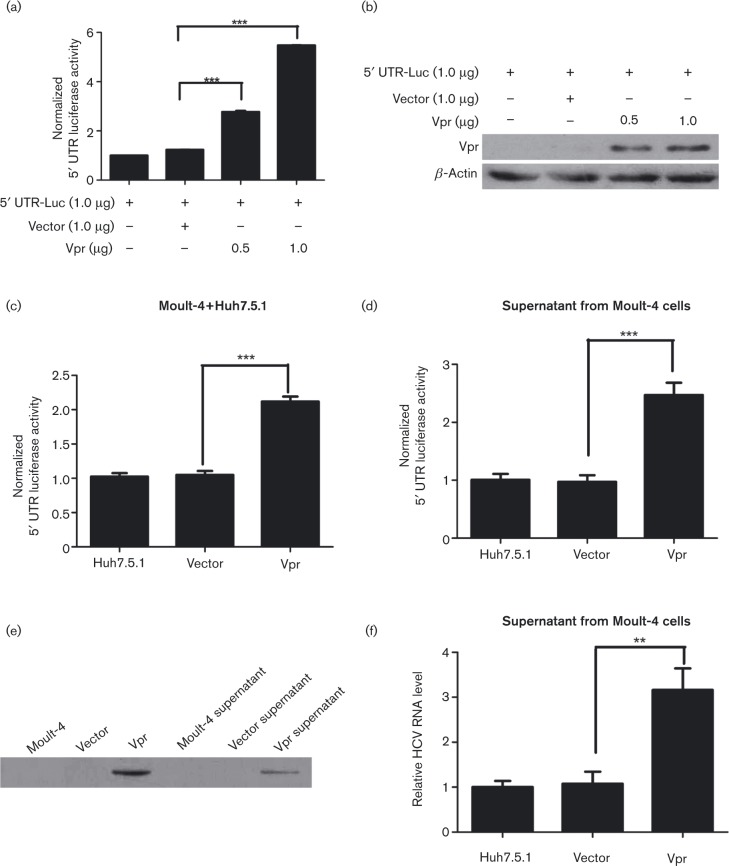
Vpr induces HCV 5′ UTR reporter activity, is secreted from lymphocytes and then activates HCV replication in hepatocytes. (a) An HCV 5′ UTR-driven luciferase (pUTR-Luc) reporter plasmid and pRL-TK expressing *Renilla* luciferase were co-transfected with the indicated plasmids at different doses in Huh7.5.1 cells for 72 h. Dual luciferase assay was performed. (b) Western blotting analyses confirmed that Vpr at different doses was transfected into Huh7.5.1 cells. (c) Dual luciferase assay of pUTR-luc in Moult-4 and Huh7.5.1 co-cultured cell lines transfected with Vpr and controls. (d) Dual luciferase assay of pUTR-luc in Huh7.5.1 cells incubated with the supernatants from Vpr-Moult-4 or vector-Moult-4 cells. (e) Western blotting analysis confirmed the expression of Vpr in the culture supernatants from transfected Moult-4 cells. (f) The relative fold change of HCV RNA levels was measured in JFH1-infected Huh7.5.1 cells incubated with the supernatants from Vpr-Moult-4 or vector-Moult-4 cells. The data are presented as mean ± sd. **P < 0.01, ***P < 0.001.

For the important function of HCV 5′ UTR in HCV replication, we then investigated whether Vpr could increase HCV gene replication by exerting positive effects on the HCV 5′ UTR. Huh7.5.1 cells were seeded into 24-well plates and subsequently co-transfected with pUTR-Luc (1.0 μg), pRL-TK (0.1 μg), and pcDNA-Vpr or pcDNA3.1 at different doses for 72 h. The results showed that the presence of Vpr significantly increased the relative luciferase activity in a dose-dependent manner, reaching a 2.24- and 4.42-fold increase at 0.5 and 1.0 μg, respectively, compared with vector-transfected cells (*P* < 0.001, Fig. 3a). The expression of Vpr at different doses was confirmed by Western blot analysis (Fig. 3b). These data demonstrated that Vpr could enhance the HCV 5′ UTR activity.

### Vpr is released from lymphocytes and activates HCV replication in hepatocytes

Previous studies have shown that Vpr displays protein-transducing properties, efficiently crossing multiple cell membranes (Levy *et al.*, 1994). Accordingly, to explore whether Vpr could be secreted from lymphocytes and then perform its biological functions in hepatocytes, we examined the effect of Vpr-Molt-4 cells on the activity of HCV 5′ UTR in Huh7.5.1 cells. The Molt-4 cells were seeded into six-well plates and subsequently transfected with pcDNA3.1 or pcDNA-Vpr plasmid. At the same time, the Huh7.5.1 cells were also seeded into six-well plates and then transfected with pUTR-Luc (1.0 μg) and pRL-TK (0.1 μg) plasmids. After 24 h, all the growth media were changed and the cells were washed three times with PBS. Then the Moult-4 cells were co-cultured with the transfected Huh7.5.1 cells for 72 h, at which point the dual luciferase activities were analysed. The analyses revealed that in the presence of Vpr-Moult-4–Huh7.5.1 co-cultured cells the relative luciferase activities were significantly increased in comparison with the vector-Moult-4–Huh7.5.1 co-cultured cells (2.12- versus 1.05-fold, *P* < 0.001), as shown in Fig. 3(c). To further evaluate whether Vpr was independently secreted from lymphocytes, Moult-4 cells were seeded into six-well plates and then transfected with pcDNA3.1 or pcDNA-Vpr plasmid for 24 h. The cells were then washed three times with PBS, and incubated with fresh medium for 48 h. At the same time, the Huh7.5.1 cells were also seeded into six-well plates and then transfected with pUTR-Luc (1.0 μg) and pRL-TK (0.1 μg) plasmids for 24 h. Next, the supernatant (500 μl) of the pcDNA3.1 or pcDNA-Vpr plasmid-transfected Moult-4 cells, grown for 48 h, was added to the Huh7.5.1 cells and incubated for up to 72 h, at which point the dual luciferase activities were measured. Consistently, the results showed that the supernatant from the Vpr-Moult-4 cells also significantly enhanced HCV 5′ UTR luciferase activities in comparison with the vector-Moult-4 cells (2.47- versus 0.97-fold, *P* < 0.001), as shown in Fig. 3(d). Western blot analyses showed that Vpr was released into the culture supernatants by Vpr-Moult-4 cells (Fig. 3e), indicating that Vpr could be secreted from lymphocytes. Next we sought to identify the role of Vpr in the JFH1 virus infection system. Thus, we also incubated the JFH1-Huh7.5.1 cells with the supernatant (500 μl) from the Vpr-Moult-4 or vector-Moult-4 cells for 72 h and measured the HCV RNA levels. The results indicated that the HCV RNA levels were significantly increased in the presence of the supernatant from Vpr-Moult-4 cells in comparison with the levels seen with supernatants from vector-Moult-4–Huh7.5.1 cells (3.16- versus 1.08-fold, *P* < 0.001), as shown in Fig. 3(f). The results showed that Vpr could be independently secreted from the lymphocytes, and then promoted the HCV 5′ UTR activity and activated HCV replication in hepatocytes.

### Vpr upregulates the expression of miR-122

**Fig. 4.**
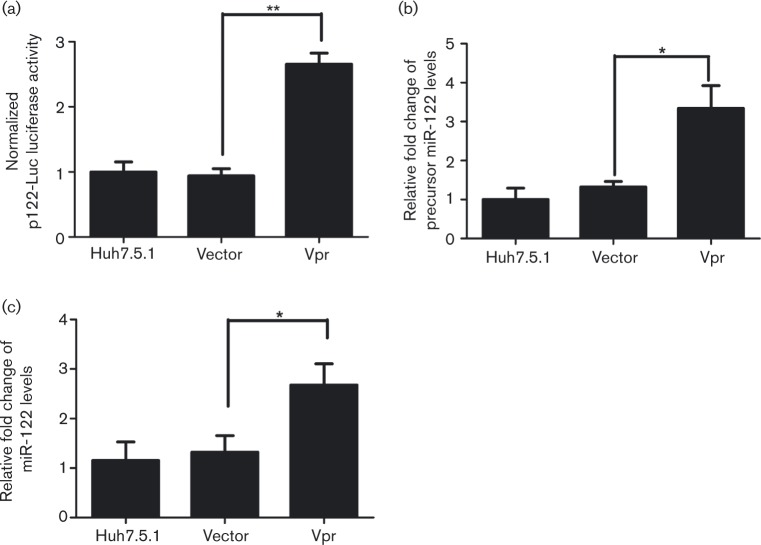
Vpr stimulates miR-122 promoter activity. (a) An miR-122 promoter-luciferase (p122-Luc) reporter plasmid and pRL-TK were co-transfected into Huh7.5.1 cells, combined with the indicated plasmids for 72 h. Dual luciferase assay was performed. (b) qRT-PCR assay of the expression levels of precursor miR-122 in Huh7.5.1 cells transfected with Vpr and controls. (c) qRT-PCR assay of mature miR-122 in Huh7.5.1 cells transfected with Vpr and controls. Data are the means ± sd of results from three independent experiments. *P < 0.05, **P < 0.01.

The above results demonstrated that Vpr acts on the HCV 5′ UTR, activates HCV RNA replication and induces HCV protein expression. Next we wanted to ascertain whether Vpr affects miR-122 expression and miR-122 influences HCV 5′ UTR activity. To this end, an miR-122 promoter luciferase reporter system (p122-Luc) was used and a dual luciferase activity analysis was conducted. The analysis revealed that Vpr significantly induced the miR-122 promoter activity, reaching a 2.99-fold increase in comparison with the vector-Huh7.5.1 cells (*P* < 0.01), as shown in Fig. 4(a). Further evaluation of the effect of Vpr on miR-122, by assessing the expression of miR-122 using real-time PCR showed that the expression of precursor miR-122 in the presence of Vpr was increased to 3.34 times the reference level in the vector control cells (*P* < 0.05, Fig. 4b) and that of mature miR-122 to 2.68 times the reference level in the vector control (*P* < 0.05, Fig. 4c). These findings confirmed that Vpr could activate miR-122 expression by inducing miR-122 promoter activity.

### miR-122 is involved in Vpr-mediated enhancement of HCV 5′ UTR activity and HCV replication

**Fig. 5.**
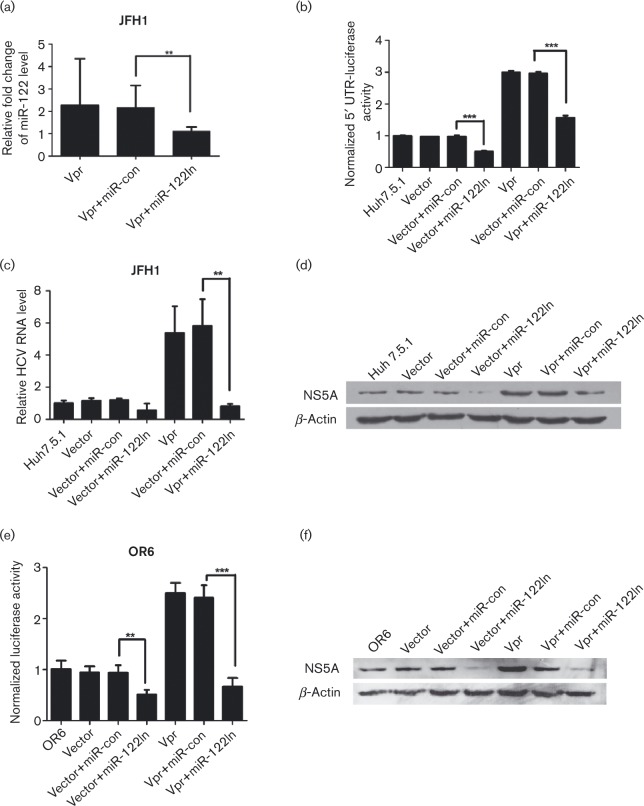
MiR-122 is essential for the effect of Vpr on HCV 5′ UTR activity, HCV RNA replication and HCV protein expression in the JFH1 infection model and OR6 cell lines. (a) qRT-PCR assay of the expression levels of miR-122 in miR-122-silence Huh7.5.1 cell lines and controls (miR-con). Results were normalized with respect to U6 mRNA. (b) Dual luciferase assay of p5′ UTR-luc in miR-122-silence Huh7.5.1 cell lines transfected with Vpr and controls. (c) Quantitative HCV RNA assay in miR-122-silence Huh7.5.1 cell lines transfected with Vpr and controls. (d) Western blot analysis in miR-122-silence Huh7.5.1 cell lines confirmed the expression of β-actin, NS5A and Vpr. (e) *Renilla* luciferase assay in miR-122-silence OR6 cell lines transfected with Vpr and controls. (f) Western blot analysis in miR-122-silence OR6 cell lines confirmed the expression of β-actin, NS5A and Vpr. The data are presented as mean ± sd. **P < 0.01, ***P < 0.001.

Our results so far have shown that Vpr activated HCV 5′ UTR, induced miR-122 expression, and promoted HCV replication, but not much is known about what their relationships are. Therefore, we assessed the involvement of miR-122 in the mediation of the effects of Vpr on HCV replication. Analysis of the expression of miR-122 by real-time PCR in the presence of miR-122 inhibitor (miR-122In), which was used to block miR-122 expression, showed that miR-122In eliminated miR-122 expression compared with the miRNA negative control inhibitor (miR-con)-treated cells (*P* < 0.01), as shown in Fig. 5(a), which indicated that miR-122 expression was successfully inhibited.

miR-122 had been reported to promote HCV RNA replication by directly binding to HCV 5′ UTR. Thus, we assessed whether miR-122 was critical for the induction of HCV 5′ UTR activity by Vpr. The assessment revealed that the miR-122In could strongly abolish the positive effect of Vpr on HCV 5′ UTR activity, which was reduced to 1.58-fold compared with that of miR-122-con-treated cells which was 2.93-fold (*P* < 0.001), as shown in Fig. 5(b). These results suggested that the Vpr-induced HCV 5′ UTR activity was closely related to miR-122 expression. Meanwhile, miR-122In exhibited a similar effect on the JFH1 infection system. Consistently, the activation of HCV RNA replication by Vpr in JFH1 replication cells was reduced to 0.81-fold in the presence of the miR-122In in comparison with the Vpr-miR-con-treated cells (5.80-fold, *P* < 0.01), as illustrated in Fig. 5(c). Western blot analysis confirmed that the miR-122In also significantly blocked the induction of the HCV NS5A protein expression by Vpr in comparison with the miR-con in Vpr-Huh7.5.1 cells (Fig. 5d).

Additionally, the effect of miR-122 knockdown was investigated in the OR6 system. The results showed that, when Vpr-OR6 cells were treated with miR-122In, the relative luciferase activity (0.67- versus 2.41-fold, Fig. 5e) and the expression of the HCV protein (Fig. 5f) were significantly decreased in comparison with the miR-con-treated Vpr-OR6 cells (*P* < 0.001).

Taken together, these results indicated that miR-122 was closely involved in the Vpr-mediated stimulation of the HCV 5′ UTR activity, HCV RNA replication and HCV protein expression. Blocked miR-122 expression eliminates the effect of Vpr on HCV replication.

## Discussion

Epidemiological studies have shown that the serum HCV RNA load is much higher in HIV/HCV co-infected patients than in HCV mono-infected patients (Beld *et al.*, 1998; Cribier *et al.*, 1995; Eyster *et al.*, 1994). The mechanisms by which HIV contributes to the stimulation of HCV replication in HIV/HCV co-infected patients have still not been elucidated. Some studies have claimed that HIV can infect hepatocytes, and the upregulation of HCV replication may be related to HIV replication in liver cells. However, other data have shown that infectious HIV failed to replicate in liver cells, indicating that maybe HIV proteins directly or indirectly influenced HCV replication. The HIV gp120, Rev and Tat proteins have been confirmed to enhance HCV replication (Lin *et al.*, 2008; Qu *et al.*, 2011, 2012), and Vpr was reported to stimulate HCV replication at almost the same time as we performed our experiment (Deng *et al.*, 2014). In the present study, our data clearly showed that Vpr enhanced HCV RNA replication and HCV protein expression in two independent HCV replicon systems, namely the JFH1 infectious system and OR6 cell model, both in transiently Vpr-expressing cells and in stably Vpr-expressing cells. In addition, we found that Vpr could be secreted from lymphocytes, diffuse into hepatocytes, and then activate HCV replication, thus providing a basis for the interaction between Vpr and HCV replication.

The HCV 5′ UTR functions as an important regulatory element in HCV replication and has become an attractive target for antiviral agents. In this experiment, we further investigated whether Vpr activates HCV 5′ UTR. Our results evidenced that Vpr induced HCV 5′ UTR-driven luciferase activity in a dose-dependent manner. These results appear to conflict with those reported by Deng *et al.* (2014), who found that Vpr had little impact on HCV IRES-mediated translation by using a dual-luciferase reporter system containing the HCV 5′ UTR and 5′ cap structure, but Deng *et al.* did not further investigate the molecular mechanism whereby Vpr enhances HCV RNA replication. However, these discrepant results may be due to the use of different 5′ UTR-driven luciferase reporter gene systems.

miR-122 plays a critical role in the HCV life cycle (Henke *et al.*, 2008; Jopling *et al.*, 2005). One of the mechanisms by which miR-122 promotes the HCV life cycle involves the ability of miR-122 to stabilize the HCV RNA by protecting its 5′ end from exonucleolytic degradation (Conrad *et al.*, 2013; Henke *et al.*, 2008; Shimakami *et al.*, 2012), while the other may involve the ability of miR-122 to enhance HCV replication by attaching to two tandem binding sequences within the HCV 5′ UTR and upregulating HCV 5′ UTR activity (Henke *et al.*, 2008; Jopling *et al.*, 2005). Moreover, in HIV-infected T-cell lines, the expression of miR-122 has been found to be significantly upregulated (Triboulet *et al.*, 2007), as illustrated by a recent genome-wide analysis of peripheral blood mononuclear cells in HIV/HCV co-infection (Gupta *et al.*, 2014). These previously reported data prompted us to investigate whether Vpr upregulates HCV 5′ UTR activity and then activates HCV RNA replication by way of miR-122. Through the use of miR-122 promoter-driven luciferase reporter plasmid, we studied the effect of Vpr on miR-122 expression in hepatocytes and found that Vpr directly stimulated the miR-122 promoter activity. The expression levels of both precursor and mature miR-122 significantly increased in the presence of Vpr. The mechanism whereby Vpr stimulates the miR-122 promoter activity may be related to the TATA box in the miR-122 promoter, which can be bound by the Vpr protein molecule (Li *et al.*, 2011). Previous study has also shown that a TATA box in the U_3_ region of the HIV LTR is necessary for the LTR transactivation induced by Vpr (Felzien *et al.*, 1998), suggesting that Vpr may bind to the TATA box in the miR-122 promoter and stimulate the promoter activity. The inhibition of miR-122 demonstrated that the miR-122In led to a significant decrease in Vpr-induced HCV 5′ UTR activity, HCV RNA replication and HCV protein expression. These results strongly suggest that miR-122 plays a very important role in the upregulation of HCV replication and HCV protein expression induced by the HIV Vpr protein. However, the miR-122In cannot completely abolish Vpr-induced HCV replication, suggesting that other mechanisms may exist. Previous studies have also shown that Vpr has an ability to cause G2 arrest. G2 arrest creates a favourable environment for maximizing virus replication (Goh *et al.*, 1998; Poon *et al.*, 1998). In addition, Vpr can act as a co-activator in the activation of other elements rather than as a direct transcription factor. It has been proposed that Vpr transactivates the HIV LTR via binding to the Sp1–promoter complex (Amini *et al.*, 2004; Sawaya et al., 1998). Additionally, the interaction between Vpr and miR-122 may be involved in binding other proteins, which form a promoter complex with Vpr to cooperatively induce miR-122 transcription.

In conclusion, our results clearly showed that Vpr stimulated HCV RNA replication and HCV protein expression by acting on the HCV 5′ UTR. Vpr was found to be released from lymphocytes, diffused into hepatocytes, and then performed its role in HCV replication. We also evidenced that Vpr upregulated miR-122 expression by activating the miR-122 promoter, followed by an increase in the expression of the precursor and mature miR-122 molecules. Furthermore, the application of miR-122In could almost abolish the effects of Vpr on HCV 5′ UTR activity, and decreased the Vpr-induced HCV RNA replication and HCV protein expression. Overall, our findings indicated that HIV Vpr stimulated HCV replication through the upregulation of the miR-122 pathway. These data provide new insight into the mechanisms whereby HIV enhances HCV replication in HIV/HCV co-infection.

## Methods

### Cell lines and HCV infection cell models

Huh7.5.1 cell lines (human hepatocellular carcinoma cells) were cultured in Dulbecco's Modified Eagle's Medium (DMEM) (Gibco) and Moult-4 cell lines were cultured in RPMI 1640 (Gibco), both supplemented with 10 % FBS (Gibco) and penicillin (100 U ml^− 1^)/streptomycin (0.1 mg ml^− 1^) (Gibco). The OR6 replicon cell line, which was cloned from ORN/C-5B/KE cells and which harbours full-length HCV RNA (HCV genotype 1b) and co-expresses *Renilla* luciferase, was cultured in DMEM supplemented with 10 % FBS, and 400 μg G418 ml^− 1^ (Biosharp). The cells were maintained in a humidified incubator at 37 °C in a 5 % CO_2_ atmosphere. JFH1 genomic RNA (HCV genotype 2a) was delivered into Huh7.5.1 cells as previously described. We seeded cells for 24 h in a 24-well plate or a 6-well plate before infection with 100 μl inoculum, and then transient transfection with the indicated plasmids. The HCV cell culture supernatant and cellular RNA were collected at the appropriate time points. The viral titre of the supernatant was expressed as international units (IU) ml^− 1^ by PCR–fluorescence probing.

### Plasmids and transfection

The HIV-1 WT Vpr-expressing plasmid, pcDNA-Vpr, was kindly donated by Professor Yuhuang Zheng (AIDS Laboratory, Department of Infectious Diseases, Second Xiangya Hospital). pcDNA3.1 was used as the vector expression plasmid. The HCV 5′ UTR was cloned from the plasmid-containing HCV 1b full-length genome, pHCV1b, kindly gifted by Professor Aleem Siddiqui (University of California, San Diego, CA, USA), and the miR-122 promoter-luciferase reporter plasmid (p122-Luc) was cloned by PCR from human genomic DNA by means of the following primers: HCV 5′-UTR, 5′-AATACGCGTACCCGCCCCTAATAGGGGCGACACTC-3′ (sense) and 5′-AATCTCGAGGGTGCACGGTCTACGAGACC-3′ (anti-sense); miR-122 promoter, 5′-CGATACGCGTGAATGCATGGTTAAC-3′ (sense) and 5′-TGATCTCGAGCCTCCCGTCATTTCT-3′ (anti-sense). The two sequences were inserted into the *Mlu*I and *Xho*I sites of the pGL3-Basic Luciferase Reporter Vector (Promega). The cells were seeded into six-well plates at a density of 3 × 10^5^ cells per well. After 24 h, the cells approaching 70–80 % confluence were transfected with individual plasmids using Lipofectamine 2000 (Invitrogen).

### Construction of a stable Vpr-expressing cell line

To establish a stable Vpr-expressing cell line, Huh7.5.1 cells were seeded in 60 mm dishes and then transfected with pcDNA-Vpr or pcDNA3.1 vector plasmid for 48 h before the initial selection of stable colonies with 400 μg G418 ml^− 1^ to the culture medium. G418-resistant colonies were maintained in medium supplemented with 200 μg G418 ml^− 1^. G418-resistant colonies of Vpr-expressing (Vpr-Huh7.5.1) and vector-expressing (vector-Huh7.5.1) cells were confirmed by PCR, Western blotting and sequencing.

### Luciferase reporter gene assay

OR6 cells were seeded into 24-well plates. After 24 h, the cells were transfected with the Vpr plasmid or vector. *Renilla* luciferase activity was evaluated using the *Renilla* Luciferase Assay kit (Promega). The pUTR-Luc (1.0 μg) or p122-Luc (1.0 μg), along with *Renilla* luciferase (RL) expression vector (pRL-TK) (0.1 μg), was co-transfected with Vpr or vector plasmid into cells using Lipofectamine 2000. After 72 h of transfection, the cells were washed with PBS and lysed with 100 μl (24-well) or 500 μl (6-well) lysis buffer. The relative luciferase activity was evaluated using the Dual Luciferase Assay kit (Promega).

### Oligonucleotides

miR-122 inhibitor (the 2′-*O*-methylated anti-miR-122 oligonucleotide, hsa-miR-122-5p, miRBase ID MIMAT0000421, inhibitor sequence CAAACACCAUUGUCACACUCCA) and a control 2′-*O*-methylated oligonucleotide (miR-con, sequence CAGUACUUUUGUGUAGUACAA) were supplied by RiboBio. JFH1-infected Huh7.5.1 cells or OR6 cells were seeded into six-well plates. After 24 h, miR-122In or miR-con (100 nM) was transfected into cells using Lipofectamine 2000 according to the protocol, and the medium containing miR-122In or miR-con with Lipofectamine 2000 complexes was replaced after 4 h of transfection. Total RNA or protein was prepared 72 h after transfection for the next experiment.

### Real-time PCR

Total RNA was harvested from cells using TRIzol reagent (Invitrogen). Using the TaKaRa One Step PrimeScript miRNA cDNA Synthesis kit (Perfect Real Time), 1 μg total RNA was reverse-transcribed into complementary DNA (cDNA). Quantitative real-time PCR (qRT-PCR) was performed with an ABI 7500 Real-Time PCR System (Applied Biosystems) using Takara SYBR_Premix ExTaqTM II (Perfect Real Time). The primers used for reverse transcription of cDNA were as follows: miR-122, 5′-GTCGTATCCAGTGCAGGGTCCGAGGTATTCGCACTGGATACGACCAAACA-3′; precursor miR-122, 5′-GCCTAGCAGTAGCTATTT-3′; U6, 5′-TTCACGAATTTGCGTGTCAT-3′. The following primers were used in the qRT-PCR: miR-122, 5′-TCGCCTGGAGTGTGACAATGG-3′ (sense) and 5′-GTGCAGGGTCCGAGGT-3′ (anti-sense); precursor miR-122, 5′-TTAGCAGAGCTGTGGAGT-3′ (sense) and 5′-GCCTAGCAGTAGCTATTT-3′ (anti-sense); U6, 5′-CGCTTCGGCAGCACATATAC-3′ (sense) and 5′-TTCACGAATTTGCGTGTCAT-3′ (anti-sense). Quantitative PCR was performed according to the manufacturer's instructions, with an initial denaturation step at 95 °C for 10 min, followed by 40 cycles of denaturation at 95 °C for 15 s, and annealing at 60 °C for 60 s. The human U6 was used as a control for basal RNA levels. The ΔΔ*C*_t_ method for relative quantification of gene expression was used to determine the mRNA expression levels. The fold change was demonstrated using the equation 2^− ΔΔ*C*_t_^.

### Quantitative HCV RNA assay by PCR–fluorescence probing

Total RNA from cell culture supernatant (200 μl) was extracted by the magnetic beads method (Sansure Biotech). The RNA pellet was dissolved in 50 μl PCR-mix, and then reverse transcription and PCR amplification were performed in the ABI 7500 Real-Time PCR System (Applied Biosystems) by using the Hepatitis C Viral RNA Quantitative Fluorescence Diagnostic kit (Sansure Biotech) with a detection limit ≥ 25 HCV RNA IU ml^− 1^ (sample type: serum, plasma).

### Western blot analysis

Proteins were resolved using SDS-PAGE and transferred onto PVDF membranes. The membranes were then blocked with 10 % non-fat milk at room temperature for 30 min and probed with the indicated primary antibodies for 90 min at room temperature or overnight at 4 °C. The primary antibodies used in this study were mouse monoclonal anti-NS5A (SC-65458; Santa Cruz Biotechnology), goat polyclonal anti-Vpr (SC-17493; Santa Cruz Biotechnology), and mouse monoclonal anti-β-actin (TA-09; ZSGB-Bio). The secondary antibodies were horseradish peroxidase (HRP)-conjugated ECL goat anti-mouse IgG (SC-2005; Santa Cruz Biotechnology) and donkey anti-goat IgG (SC-2020; Santa Cruz Biotechnology).

### Statistical analysis

Data are expressed as mean ± sd of at least triplicate experiments, unless stated otherwise. Significant differences (*P* values), unless otherwise indicated, were analysed using one-way ANOVA. A *P* value < 0.05 was considered significant.
